# Therapeutic Itineraries, Therapeutic Itinerances in Democratic Republic of Congo

**DOI:** 10.29245/2578-3009/2023/S3.1101

**Published:** 2023-05-12

**Authors:** Tieman Diarra, Joseph Okeibunor, Amadou Baïlo DIALLO, Nkechi Onyeneho, Barry Rodrigue, Michel N’da Konan Yao, Zabulon Yoti, Ibrahima Socé FALL

**Affiliations:** 1 Independent Consultant, Mali; 2 World Health Organization, Switzerland; 3 University of Nigeria, Nsukka

**Keywords:** Therapeutic itinerary, Therapeutic roaming, Self-medication, Traditional medicine, Modern medicine, Perceptions of disease, Treatment, Healing, Ebola survivors

## Abstract

While treating a disease, patients or their relatives make decisions to pursue different therapeutic options, and various stages are involved in searching for a cure. This paper explored the pattern of health-seeking in the Democratic Republic of Congo (DRC) during the 10th Ebola virus disease (EVD) outbreak. Eight hundred randomly selected adults were surveyed using a questionnaire. Qualitative data were also collected through in-depth interviews with 17 community leaders and 20 focus group discussions with community members. The results showed that modern healthcare facilities are not usually considered the first option for treatment. The therapeutic journey generally begins with the patients, who treat themselves based on the what they know about the disease and the resources they have at their disposal. However, if the disease is not cured through self-medication, then patients or their relatives will visit a pharmacy. Patients request medication they know to be effective in treating the disease, and relatives can also assist in obtaining medication in the case of immobile patients. Pharmacies commonly sell the medication to patients or their relatives without a medical prescription.

## Introduction

Patients and their relatives have many therapeutic choices on the road to recovery, and many decisions must be made to determine the best course of action for treating a disease; however, these decisions are usually made in different stages^1,2^. They usually start with the patient self-medicating, which is the easiest option, as hardly any additional resources need to be devoted to this method, which is carried out with medication on hand. Patients use the information they already know about the disease and the medication available to them to treat it.

When self-medication does not lead to a cure, patients then turn to a pharmacy for assistance—this is usually done without a prescription^3,4^. If the medication patients obtain from a pharmacy does not lead to a cure, then the patients or their relatives generally seek assistance from a traditional healer. If a traditional healer is unable to treat the disease, only then do the patients or their relatives consider seeking treatment from a modern healthcare center. Frustration concerning failed attempts at a cure drives patients or their relatives to consider other options for treatment. Healthcare-seeking behavior is guided by the resources available to the patient for dealing with the disease and varies according to the proximity of a healthcare center. This proximity is not only geographical but also sociocultural. For instance, often traditional healers live in the patient’s community or a nearby community, and thus usually have a lot in common with them.

The result achieved at the healthcare center will affect the patient’s decision to seek further care, if necessary. If the treatment received at the nearest healthcare center is not satisfactory, the patient or their relatives will seek a referral to a larger healthcare facility and then a hospital. This choice is guided by their concerns for finding better treatment options and increasing the likelihood of returning to a normal state of health. A hospital will usually offer the greatest possibility of recovery. Our interviews with patients and modern healthcare providers found that some people may resort to seeking healthcare from a hospital as a first option, but this is not the norm in the provinces of North Kivu and Ituri in the Democratic Republic of Congo (DRC).

The symptoms of the disease also affect patients’ choices made concerning treatment, and people do not generally seek healthcare from a modern medical facility for diseases that are considered to fall exclusively within the domain of traditional medicine^5,6^. The therapeutic journey does not always have a straight and direct path, and patients may resort to using modern medicine, traditional medicine, and self-medication in any order and at different stages of treatment. Patients and their relatives may use one method and then try another one, only to go back to the initial method used. A therapeutic itinerary may thus entail a great deal of therapeutic roaming. It appears that a therapeutic itinerary is used when patients want to try everything in their power to increase their chances of recovery. Nevertheless, this therapeutic pluralism is potentially dangerous regarding the Ebola virus disease (EVD) as it potentially increases the chances of transmitting the disease.

This study aimed to evaluate the therapeutic itineraries of those with Ebola in the provinces of North Kivu and Ituri in the DRC and its polymorphism. We also aimed to assess the transformation of the therapeutic itinerary into therapeutic pluralism.

## Study Design and Methods

### Study design

This study was designed to explore and document experiences and lessons around the response to the 10^th^ EVD outbreak in the North Kivu and Ituri provinces of the DRC. It adopted a cross-sectional design with mixed-method techniques of data collection. The design allowed multiple windows of data harvesting, while the mixed-method approach brought the benefits of both quantitative and qualitative methodologies and guaranteed the integrity and robust interpretation and findings of this study.

### Selection of the study area and population

The study was carried out in North Kivu and Ituri provinces of the DRC, where the 10^th^ EVD outbreak occurred.

**Ituri** is one of the 26 provinces of the DRC. Its capital is the city of Bunia. The Ituri Rainforest is in this area. It is located northeast of the Ituri River and on the western side of Lake Albert. Ituri is a high plateau region (2,000–5,000 m) that has not only a large tropical forest but also a savannah landscape. The district has rare fauna, including the okapi, the national animal of the Congo. Concerning flora, an important species is the Mangongo, whose leaves are used by the Mbuti to build their homes. Ituri is inhabited by different populations of Alur, Hema, Lendu, Ngiti, Bira, and Ndo-Okebo, and one group constitutes the largest percentage of the total population. The Mbuti, a pygmy ethnic group, reside primarily in the Ituri forest near the Okapi Wildlife Reserve; however, some of them have been forced into urban areas by deforestation, overhunting, and violence. The Kilo–Moto gold mines are partly located in Ituri where, at the start of the 21st century, petroleum reserves were found by Heritage Oil and Tullow Oil on the shores of Lake Albert.

**North Kivu** (French: *Nord-Kivu*) is a province bordering Lake Kivu in the eastern DRC. Its capital is Goma. North Kivu borders the provinces of Ituri to the north, Tshopo to the northwest, Maniema to the southwest, and South Kivu to the south. To the east, it borders the countries of Uganda and Rwanda. The province has three cities: Goma, Buttembo, and Beni, and six territories Beni, Lubero, Masisi Rutshuru, Nyiragongo, Walikale. The province is home to the Virunga National Park, a World Heritage Site containing the endangered mountain gorillas. Except for the heightened insecurity and isolation due to rebel activities, North Kivu shares similar demographics with Ituri. The province is politically unstable and has been one of the flashpoints of the military conflicts in the region since 1998.

The **2018 or 10^th^ Kivu Ebola outbreak** occurred on August 1, 2018, when four individuals tested positive for the Ebola virus in the eastern region of Kivu in the DRC^7,8,9^. The Kivu outbreak included the Ituri Province, after the first case was confirmed on August 13, 2018^10^. This outbreak started just days after the end of the 2018 Équateur province Ebola virus outbreak in the DRC^11,12^.

The affected province and general area were under a military conflict, which hindered treatment and prevention efforts. The World Health Organization’s (WHO) deputy director-general for emergency preparedness and response described the combination of military conflict and civilian distress as a potential “perfect storm” that could lead to a rapid worsening of the outbreak^13^. Owing to the deteriorating situation in North Kivu and the surrounding areas, the WHO, on September 27, 2018, raised the risk assessment at the national and regional levels from “high” to “very high”^13^.

The study population comprised adults aged ≥ 18 years living in the community, and response team members. A 2010 estimate put the population of North Kivu at 5,767,945. With an annual growth rate of 3.2%, the population in North Kivu in 2019 was estimated at 7,658,406 and 5,360,884 for the general and age ≥18 years populations, respectively. Meanwhile, a 2005 estimate put the population of Ituri at 4,037,561, and an estimate of the population aged ≥ 18 years at 74% resulted in 2,968,865 individuals. For 2019, the populations were estimated as 6,275,305 and 4,392,714 for the general and age ≥18 years populations, respectively.

The response team consisted of over 10,000 persons, comprising different response pillars, namely, surveillance, risk communication, social anthropology, and vaccination. Others included infection prevention and control, treatment and care, safe and dignified burial, security, logistics, and administration.

### Sample size estimation and sampling strategy

#### Sample size

Although this was an exploratory study, to achieve statistical conclusions on certain indicators of perceptions and practices—juxtaposed with relevant demographic characteristics—a sample of the study population was taken. With an assumed 50% chance of having accepted Ebola control interventions at a confidence interval of 95% and an error margin of 5%, a sample size of 384 was computed for the quantitative study. For the two provinces, the sample size was calculated at 768; this number was rounded up to 800 to allow for losses. The size of the qualitative study depended on the saturation of information after two sets of data were collected from each category of respondents.

#### Sampling strategy

A multi-stage sampling technique was adopted in selecting the communities, households, and respondents for this study. Two administrative areas (that are epicenters of EVD outbreaks within each province) were purposely selected. Ten communities were randomly chosen from each of the two administrative areas in the province.

#### Selection of households and respondents

The center of the selected community was used as our reference point to randomly determine the first route and first household of our sample. This was done by spinning a pencil from the center. Thereafter, we made our selections by moving to the right to identify the next household. This continued until we reached the required number of households to be sampled. Where there was a *cul-de-sac*, the step was retraced, and a turn to the left and then to the right was made to continue the sampling process.

One adult ≥ 18 years was randomly selected from each chosen household for inclusion as a study participant. the sex of the participants was carefully alternated—that is, where in household number one, a male was selected, in the next, the focus was to select a female.

## Methods

The study was conducted using a mix-method approach of qualitative and quantitative techniques. The methodology for data gathering involved in-depth interviews, focus group discussions (FGDs), and a survey using structured questionnaires. This type of study requires a strong focus on individual actors rather than state actors^14^.

## Techniques of data collection

**Focus group discussions** (FGDs): These were distributed in North Kivu as shown in Table 1. The same distribution was actualized for Ituri province.

A set of questions covering different thematic areas was developed to guide the discussions. The questions covered health care services in the community, awareness of and practices on EVD, and an assessment of the different pillars of the response interventions.

For the FGDs, 8 to 12 individuals were selected for each session. A minimum of two FGDs were conducted in the selected communities. Separate FGD sessions were held for men and women in each of the communities. Overall, eight FGD sessions were conducted in each province.

**In-depth interviews (IDIs)** were conducted in each community where FGD was carried out. The IDIs were held with community/opinion leaders in the selected communities and the team leaders of the response pillars. Interviews were used to explore people’s opinions, views, and attitudes as practices and insights into the outbreak and response and other sociocultural factors that may influence the attitude toward the response. The FGD guide was used for the in-depth interview, focusing on the thematic areas of interest of the evaluation.

**A structured questionnaire** was used to collect quantitative data from households. The questionnaire incorporated all the indicators that were used to answer the research questions and was structured based on the qualitative study results. It was categorized into the following sections: socio-demographic data, perception of health problems in the community, knowledge of EVD, perceived epidemiology of Ebola in the communities, and sources of information on Ebola. The questionnaire also covered issues on communication and community engagement, infection prevention and control in the communities, vaccination, surveillance, and treatment and care. Other sets of questions covered the topics of a safe and dignified burial, psychosocial issues, logistics, and security issues.

All interviews and discussions were tape-recorded, and detailed notes were taken simultaneously, including verbal citations. Tape-recorded interviews were transcribed according to standard rules. Observations were also recorded and, together with discussion and interviews, triangulated using the quantitative data to arrive at conclusions.

### Training and pilot trials

All instruments were ***translated*** into Swahili and French, the common languages spoken in the communities, and then translated back into English for clarity. In each province, we recruited ***10 research assistants*** with substantial experience in community interactive research who were familiar with qualitative and quantitative techniques and cultural sensibility. They trained for three days in Beni and another three days in Bunia on the study objectives and the use of the instrument for data collection. Training also included data entry into the Atlas.ti template (for qualitative data) and EPI INFO (for quantitative data). The instruments were reviewed after training, for clarity, understanding, and sensitivity. Each province had a ***supervisor*** who worked with the principal investigator on data quality monitoring, safety advisory, and the ethical conduct of the research, including the management of informed consent procedures. The study was conducted first in Ituri, and then in North Kivu. The lessons learned from Ituri were used to manage the process in the North Kivu area, which is a more challenging province regarding security and logistics. The ***data analyst*** developed and pre-tested the template for data entry and analysis using the pilot test output. Given the short study period, data were collected using pencil and paper instead of android devices. The fieldwork took 20 days to complete for each province before the analysis and report writing were conducted.

### Data management

All **quantitative data** were double-checked by the researcher before computer input. Data were entered into EPI Info and processed using SPSS. Descriptive statistics were used to determine the proportions of various categories of respondents and indicators and for comparison. Frequency tables and graphic illustrations were used to present the data.

**Qualitative data** consisting of FGDs and IDIs were transcribed from audio records to text. All textual data were analyzed using Atlas.ti software package. Data were analyzed according to themes corresponding to the indicators in the quantitative data and triangulated during the presentation to enable a complementary and analogous interpretation.

Given the continuous analytical process involved in qualitative analyses, it is important to note that the initial analysis of the key informant interviews and FGDs informed the final development of the structured questionnaire to be used in the study. This further enhanced triangulation between the two sets of data to be collected. The quantitative results gave us statistical conclusions, whereas the qualitative results emphasized what was said and provided illustrative quotes that gave context and depth to the quantitative results.

### Ethical considerations

The principle of do-no-harm was adhered to in the study. Informed study approval was obtained from the province, local administration, community, and household, while informed consent was obtained from all individuals involved in the study. The WHO/AFRO Ethics Review Committee approved this study. All researchers attended the mandatory training, which included substantial discussion on relevant ethical issues. About 50% of the research assistants were women, which helped ensure same-sex interviews and moderation of FGD sessions. The assistants were also trained and mandated to comply with child protection and gender sensitivity during data collection and visits.

## Results

Our results were based on the content analysis of the interview transcriptions and FGDs and the analysis of the survey responses.

### Therapeutic itineraries do not support the use of healthcare facilities

From an analysis of the interviews conducted and the FGDs held, a classic therapeutic itinerary can be said to occur as follows:

- Self-medication
- Use of non-prescription medication obtained from a pharmacy
- Use of a traditional healer
- Use of the nearest healthcare center
- Use of a referral healthcare facility
- Use of a hospital.

We found that the sequence described above is widely established in the provinces of North Kivu and Ituri. However, this pattern is not incompatible with certain specific healthcare-seeking behaviors. Indeed, according to healthcare professionals, some people do immediately seek medical help from a healthcare center or hospital. This was found to be true for approximately one-third of the participants. This finding may have been the result of the interaction between the participants and the interviewers, who were likely to be perceived as healthcare employees. Thus, the participants’ answers were likely to be guided by the perceived status of their interviewers.

The first steps of the itinerary above are often motivated by a lack of means, and we found that the choice of treatment options depended on the extent of the patients’ available resources. Healthcare centers that offered free treatment experienced an increase in attendance, even though on average, the EVD epidemic led to a drop in attendance at healthcare facilities. Rumors about free healthcare—as a way of attracting the public and encouraging more people to get tested for EVD—positively affected attendance at healthcare centers.

Therefore, the first steps in the itinerary are usually the least expensive. In the first two stages, there is no consultation or healthcare service provider. Even in the pharmacy, the pharmacist’s opinion is not necessarily considered by the patient or their relatives when they request a particular medication. Some people go to the pharmacy with an empty box of a specific medication and then ask for a new box. However, sometimes, the pharmacist’s opinion regarding what medicine to purchase is requested after describing the patient’s symptoms.

Almost all the interviews revealed that healthcare centers are not the patients’ or their relatives’ first choice when they fall ill; instead, most will try to self-medicate before seeking outside help. Thus, our interviews revealed that self-medication was people’s first choice. This is also seen when people buy medication from pharmacies without a medical prescription. Self-medication is, in most cases, the first step, and treatment begins with self-medication at home. Pharmaceutical products, including plants and animal products, are used for self-medication, and it is only common when self-medication fails that people resort to healthcare providers (modern or traditional). In this sense, the *nganga* (a traditional healer in most Central African countries) is important. They can save human lives, unveil misdeeds, and suppress misfortunes. Some individuals may even consider the *nganga* a sort of sorcerer.

People’s first option in their disease treatment plan was also discussed with healthcare providers, as they have considerable experience with patients. From these interviews, it appears that self-medication is people’s first choice, followed by visiting a pharmacy to purchase medicine even without a medical prescription, and then resorting to traditional medicine, and lastly, to modern medicine.

Our findings revealed that some patients were sent to the Ebola Treatment Centre (ETC) in Beni. A traditional healer from Vuvatsi, not far from Butembo, reported that patients stopped seeking his help after they saw devices for transmission prevention of the disease and triage that they considered to be modern medical practices.

Another traditional healer in Mblogu, in the Rwampara health area, discussed how long patients would wait before coming to see him after starting to feel ill. He stated that some would only come five days after first showing symptoms. Moreover, he stated that he would then try to assess whether he would be able to treat the patient or if he should refer them to another traditional healer. He confirmed that some diseases are easier to recognize than others (e.g., hemorrhoids or sexual impotence). Another traditional healer in Beni referred to a divinatory diagnosis to find out if a disease fits the realm of his competence. He also claimed that patients came to him after having attended healthcare facilities. Despite our findings, many traditional healers stated that they are the patients’ last resort after having exhausted all the other available options, which conflicts with our data.

Our interviews with healthcare workers also revealed interesting insights into patients’ patterns of healthcare use. A nurse at the Ngezi Health Centre stated that when people are ill, they first go to the pharmacy, while a nurse from another healthcare center in the same neighborhood claimed that patients come to see them immediately. She reported that some patients might delay visiting a healthcare center for two days up to a week as they are likely treating themselves at home, either with the help of traditional medicine or with medication from pharmacies. She noted that she believed people took their time to go to healthcare centers owing to a lack of means. This observation was also made by almost all the healthcare workers we interviewed. Aside from the lack of financial resources, the physical distance to be travelled to get to a healthcare center was also mentioned as a constraint for patients. While explaining why people do not use these services, a nurse at the Mblogu Health Centre stated that in addition to financial problems, most people are afraid to visit healthcare facilities.

The distance to the nearest healthcare facility was an element mentioned in many interviews with the villages’ public and religious leaders in both North Kivu and Ituri. Some participants advised that the distance between their home and the nearest healthcare center was too far, while others admitted that they were close to a healthcare facility, having one (or more) located in their village. One of the residents of a neighborhood in Bunia, DRC, reported that although he lived close to a healthcare center, he preferred to visit a pharmacy first to obtain medicine to try and treat the disease. Only if there was no improvement in his condition would he then seek treatment at a healthcare center.

A nurse in charge of the Makasi health area in Butembo noted that some patients sought treatment early, but some preferred to self-medicate at home first and only make use of healthcare centers if the self-medication was unsuccessful. Moreover, she noted that many patients consider the global cost of using a healthcare center—including consultation fees, treatment, and medication costs—and opt to go to the generally less costly traditional healers. A nurse at the Boikene Health Centre expressed that the EVD outbreak had affected the use of healthcare facilities. In addition, the nurse claimed that, at the beginning of the epidemic, patients refused to seek treatment at healthcare centers or hospitals as they believed they would be infected with EVD and die there.

Rumors and misinformation affected patients’ attendance at healthcare centers during the epidemic. A resident of Ngadi said that he used herbs to treat himself for poisoning and would only go to the healthcare center for other diseases. He noted that when it comes to poisoning, patients must treat themselves with plants or herbs or seek help from a traditional healer. A woman from the same neighborhood told us that when people get sick, they self-medicate at home. When self-medication does not work, they go to a health center.

At PK 11 a village resident told us that he had hardly been to a healthcare center, even though when we interviewed him, he had already been suffering from an illness for a while.

However, he stated that he was not averse to modern medicine and the interview ended with him disclosing some private information that was not covered in our questions.

A religious leader from the same village highlighted the difficulties concerning diseases among community members if there are no pharmacies or healthcare centers in the area. As such, members of the general public who had healed from Ebola rarely started their therapeutic itinerary with modern medicine. However, we found that healthcare workers who were cured had started their treatment journeys at healthcare centers. Among the healed people we met, who were not healthcare workers, only one had chosen to visit a healthcare facility as the first treatment-seeking step. When he became ill in a village 21 km away from Mombasa, he first went to a healthcare center at PK 15. After the first treatment, he went to his family in Mombasa. He then went to the general hospital in Mandima, which recommended he go to the ETC in Mombasa.

A healed member of the medical staff told us about the use of traditional healers, which they did not believe to be necessarily inexpensive. A religious leader from Ngezi spoke of the use of a pharmacy without a medical prescription. He also noted that patients would resort to healthcare centers when self-medication does not lead to the desired result. A religious leader from Katanigwa, in the Rwampara health area, also noted that people start their treatment at home and, if that failed, then they would consider other alternatives.

A leader of PK 12 village in the same health area listed the diseases for which modern medicine is never used and even noted some diabolical diseases and poisoning. A resident of a village in the Beni area said that modern medicine would not be his first choice and that he would start at the pharmacy and would self-medicate as a means to save money.

In the FGDs, everyone agreed that when it comes to diseases, the healthcare center and the hospital are not patients’ first options. Self-medication comes first, and then they will either resort to a pharmacy or traditional medicine. Healthcare centers and hospitals are only used when previous therapeutic decisions fail.

In a discussion with a group of women in the Mabolio district of Beni, self-medication was presented as the primary means of treatment, especially for children. Overall, 51.9% of the population relies on self-medication^16^. In the Bataluka neighborhood, the same care-seeking behavior was described.

Self-medication is the first step of the therapeutic journey in many African communities. In Cameroon, a study on the determinants of self-medication shows that this is the first step in the local therapeutic journey. It is followed by the use of modern medicine and then traditional medicine, whether in urban or rural areas, or for men or women from poor, very poor, average, wealthy, or very wealthy households. Self-medication is also the first choice regardless of individuals’ level of education. The influence on the practice is mainly determined by region and it is most common in the south of the country. Access to healthcare is also a problem, which influences people’s decisions when they become ill, as access is not equal for all.

In a discussion with a group of men in Mblogu, it was highlighted that the lack of means is one reason people choose home remedies. According to this group, going to a healthcare center or a hospital is only considered when self-medication or traditional healers fail. They mentioned three well-known traditional healers in the village.

The lack of resources may also imply that people do not resort to any kind of treatment. Research carried out in Cameroon reported such a finding, described as “therapeutic abstinence.” It was observed in 3.9% to 8% of all cases^16^. In poor and extremely poor households, these numbers reached between 7.0% to 8.0% of cases. However, in most cases, people do act when they have a disease. In a group discussion in Bembo, it was unanimously agreed upon that most of the population self-medicates before going to a pharmacy or seeing a traditional healer. The healthcare center and the hospital are only considered options if the disease persists. In another discussion held in the same area, the pharmacy was also mentioned as the first choice in the case of a disease.

In Katia, in the health zone of Bembo, emphasis was placed on self-medication, the use of traditional medicine, and medication obtained from the pharmacy. It was further stated that people will resort to the pharmacy when the disease is not considered serious. In a discussion with men in the same village, self-medication with plants, followed by treatment with medicines obtained from the pharmacy, were highlighted. They explained that people go to healthcare centers only when they have the money to do so. Here, it was found that traditional healers are also one of the first options to be used.

In a discussion with a group of women from the same village, the healthcare center was not considered the first resort for most community members in the case of disease, but it was also not excluded from their therapeutic choices. However, in this group, the participants unanimously stated that if a patient is diagnosed with EVD, the hospital and the ETC are the most appropriate facilities. One participant reported that while patients should not be afraid of the hospital, they are because of Ebola. These fears are often rooted in rumors about the disease and its treatment. During the discussion, the participants mentioned 15 rumors that they had heard regarding the disease. Notably, the discussions with the participants took place in a village that was hostile to the *riposte* and to our team, which was not accepted by the local citizens. Through hard work, the team responsible for risk communication and community engagement managed to foster our acceptance by the community. In addition, they were able to transform the community’s perception of hospitals and ETCs. This community now sees hospitals and ETCs as the most appropriate places to treat Ebola. In addition, both men and women are now aware of the preventive measures needed to fight EVD.

### From Therapeutic Itinerary to Therapeutic Itinerance

The opinions expressed in the FGDs converged from one village to another, and from one town to another. It appears that self-medication is, indeed, the first step in the therapeutic process. It also emerged from the discussions and interviews that different treatments are often simultaneously used, namely, modern medicine with traditional medicine, and home plant medicines and medicines obtained from the pharmacy. Thus, it can be seen that one therapeutic choice does not exclude the use of another before, during, or after it. Therefore, we concluded that the therapeutic pathway is not always linear and the therapeutic journey often becomes therapeutic roaming.

We put together 10 focus groups, five with men and five with women. Altogether, 104 people (52 men and 52 women) participated. Research assistants conducted 80 FGDs; two in each village conducted throughout this investigation. The points highlighted above summarize what was discovered in the discussions regarding people’s therapeutic choices.

The interviews followed the same procedures. These were held with village chiefs, neighborhood chiefs, avenue chiefs, street chiefs, modern and traditional medicine providers, religious leaders, other community leaders, contacts, the healed, and administrators (territorial administrators, mayors). The results of these interviews are summarized in the diagram below, considering data from both qualitative and quantitative surveys.

The surveys show that the healthcare center and the hospital are the first places people turn to in case of disease. This contradicts our earlier findings, and we surmise that our presence and that of the research assistants influenced people’s answers. We believe that the people we interviewed may have been inclined to respond that healthcare centers or hospitals were their first choice as they felt that this was the answer we were expecting. In other words, we postulate that they would have followed an answering logic according to the expectations of those in charge of the survey. If, under these circumstances, self-medication appears in second place among the primary responses to a disease, we agree that this can be considered the dominant recourse exposed through the interviews. In the context of this triangulation, we found that the information collected by the qualitative method was predominant. We considered the statements of the healthcare personnel and traditional healers, claiming that the patients or their relatives reached out to them after having tried self-medication at home. Few healthcare providers stated that patients came to them first. Modern medicine providers generally claimed that patients came to them after having tried everything else and only after the disease became more serious and disabling. In addition, in some cases, patients would go directly to the hospital without going through healthcare facilities that would have referred them to a higher level within the healthcare pyramid.

**Figure 1:**
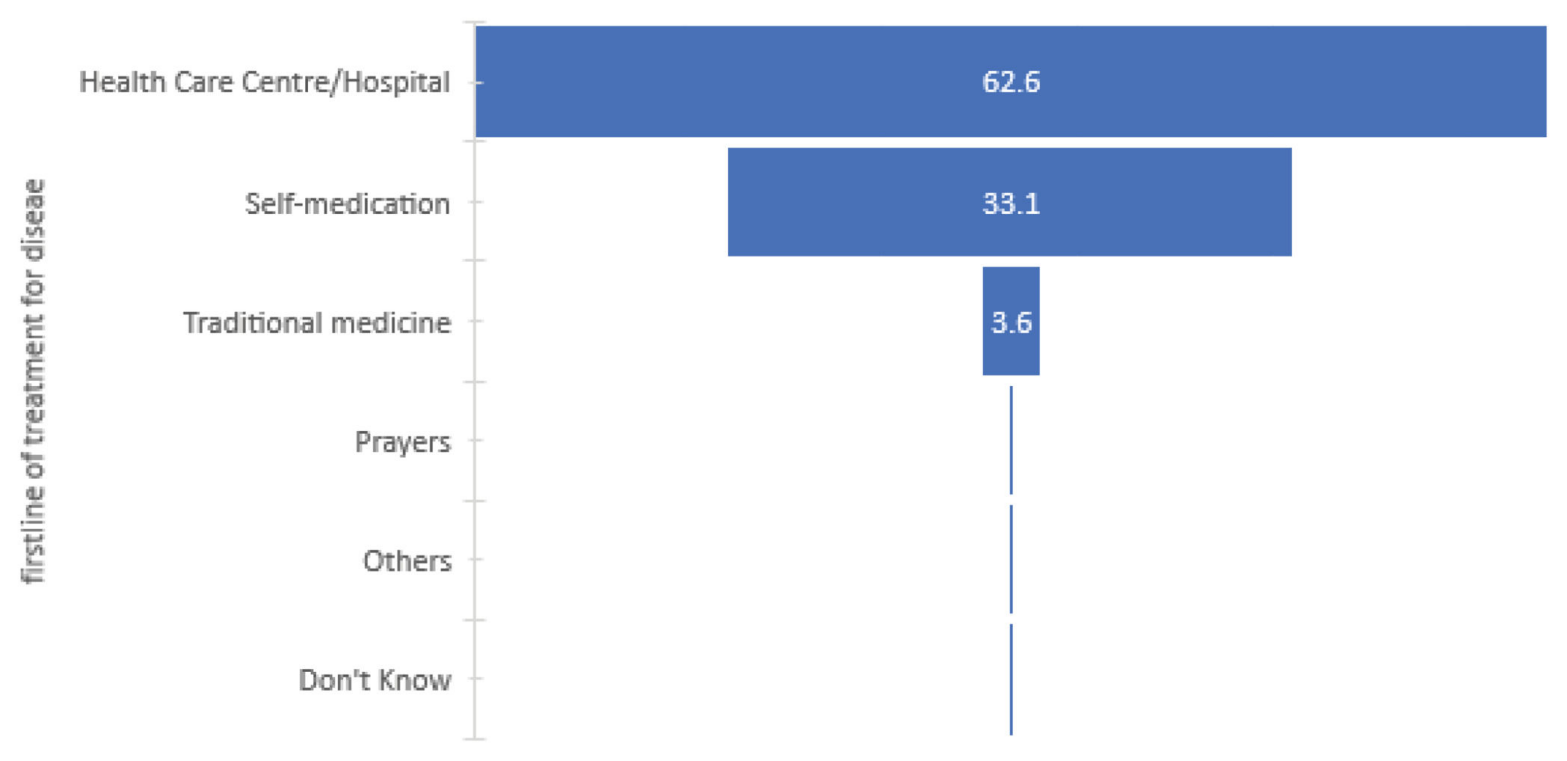
First line of treatment for a disease.

Figure 1 reveals that while most of the people interviewed stated that they first went to a healthcare center or hospital in the case of a disease, a third of them reported treating their disease through self-medication. Although the pharmacy option is not featured here, it was mentioned almost everywhere in the interviews. This omission may be true because participants provided our research team with responses according to what they thought we wanted to hear.

**Figure 2:**
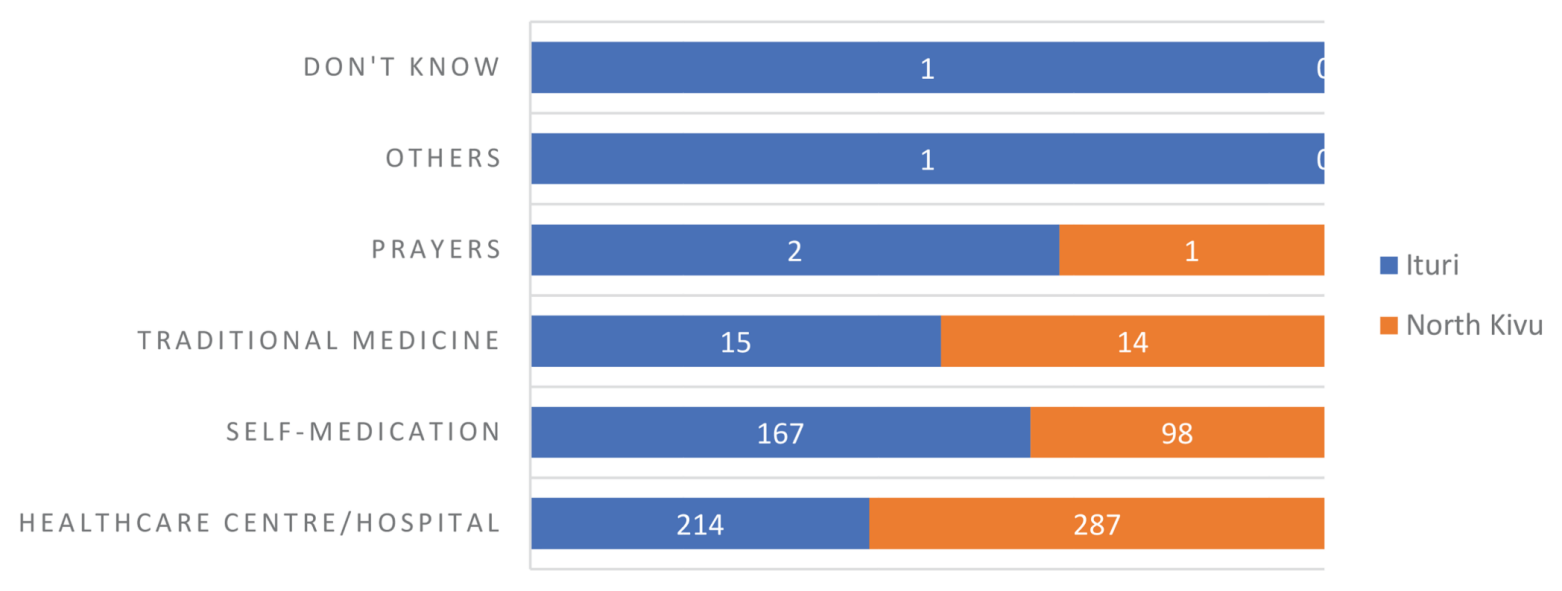
First recourse in case of a disease (by province)

Figure 2 shows that self-medication is the first option for treatment in the Ituri province for 63% of the cases, contrasting 37% in the North Kivu province. There is greater recourse to traditional medicine in the Ituri province, whereas in the North Kivu province, the healthcare center and the hospital are cited as the first option for treatment in the event of a disease. The results revealed a statistically significant difference in the reliance on the therapeutic trajectories between the two provinces, with a chi square value of 22.111 (p<0.001).

When questioned about how diseases that cannot be treated by modern medicine are cured, 505 of the 506 participants stated that traditional medicine would be appropriate. Of the 505 participants, 58.4% were from the Ituri province, and 47.1% were from North Kivu province (χ^2^ = 45.852; P<0.001). Furthermore, the participants considered EVD a demonic disease; thus, their choice of treatment also depended on their perception of the disease. They considered some diseases the exclusive domain of traditional medicine.

Approximately 61.5% of the respondents indicated that modern medicine is not considered a treatment option for some diseases. This leaves just over one-third of the sample viewing the situation differently. The outcome of this survey was almost the same in both provinces, with 57.1% of the respondents in the Ituri province answering “yes” versus 42.9% reporting the same in the North Kivu province. The difference was not significant, with an X^2^ of 4.645. Moreover, no significant differences were found between participants from rural and urban areas or their level of education.

Some of the diseases perceived to be manageable with modern medicine were poisoning, sexually transmitted diseases, sexual impotence, epilepsy, cancer, cough, and leprosy. Co-morbidities of these diseases were also suggested, especially combinations of leprosy, epilepsy, cancer, asthma, diarrhea, HIV/AIDS; and poisoning and sexual impotence. For these diseases, people believe that traditional medicine is the most appropriate treatment. Many people claimed that these diseases have the same symptoms as EVD and have cited experiencing poisoning and diarrhea when infected. When questioned about why they did not seek modern medicine as the first treatment option, two main reasons were given. Almost half of the interviewees (49.8%) answered that modern medicine does not cure diseases, and 35.4% answered that traditional remedies relieve and cure diseases. For such diseases, some spoke of self-medication as the appropriate remedy (4.5%).

## Discussion and Conclusions

We found that the population we interviewed often only perceived recourse to a healthcare facility as a necessary step when close to a patient’s death. This was found in three expressions that were used by the participants to describe what happens to the patient: “*Erimuheka; Erimwandaghalia; bakayamwitha*,” which means “to carry him, to bring him down, to kill him.” The name of the healthcare facility where the patient is taken is usually indicated in the expression. Moreover, these expressions suggest that patients and their relatives wait until the disease has progressed to an advanced stage. For instance, patients being transported likely because they are no longer mobile. The expression suggests that patients cannot get out of a vehicle and need to be carried.

The tone of the expression suggests that the only prognosis considered is death. However, it appears that death is presented as if it were the fault of the healthcare facility; that they did not treat, but rather killed the patient. This expression is reinforced by a certain perception and representation of healthcare services and does not encourage the use of a healthcare facility. Our recommendations regarding the representations of treatment facilities such as the ETC, which constitute a breeding ground for this journey toward death, are as follows.

The journey is perceived as thanatological, rather than therapeutic. The nature of the journey is also determined by the characterization of the disease. In some communities, when a disease is considered simple or natural, individuals use modern medicine, and the disease is even called a “hospital disease.” When the disease is complex (i.e., supernatural or mystical) individuals often use traditional medicine^15–18^. This determination of the cause or nature of the disease affects treatment selection^19^. An individual may turn to a traditional healer for help with diseases considered supernatural or caused by an evil spirit^20^.

Representations of the use of healthcare and treatment options change over time and according to events that take place. At the time of this study, according to a community leader, because people were returning from the Katwa Transit Centre alive, the community accepted the arrangements put in place in the Kyondo health zone. He stated that those who visited health facilities were no longer afraid of the triage system, but were afraid of being cared for by the healthcare system and were also worried about the transmission of diseases in these facilities.

Furthermore, individuals turn to churches and religious leaders, as prayer is considered curative. Therefore, churches play a significant role in health care, including in the EVD epidemic context^2^. Patients also use traditional and complementary medicine^6^, as such, many options in various configurations are used in patients’ healing journeys. It should be noted that individuals cured of Ebola benefit from a follow-up program, however, some people cannot resort to a health professional, and this is true for other diseases as well. Sadly, these people are the majority. As such, some people practice self-medication with modern medication or treat themselves with traditional medicine while some use both modern and traditional medicines^6^. Often, these choices are made to treat other diseases following their EVD recovery.

With the introduction of free healthcare, healthcare facilities have seen an increase in attendance frequency. A healthcare worker said: *“The proximity of the healthcare center also plays a major role.”* A healthcare worker at the Rwampara General Hospital said patients mainly come from nearby villages. Patients move between different therapeutic spaces as shown in the diagram below.

**Figure:**
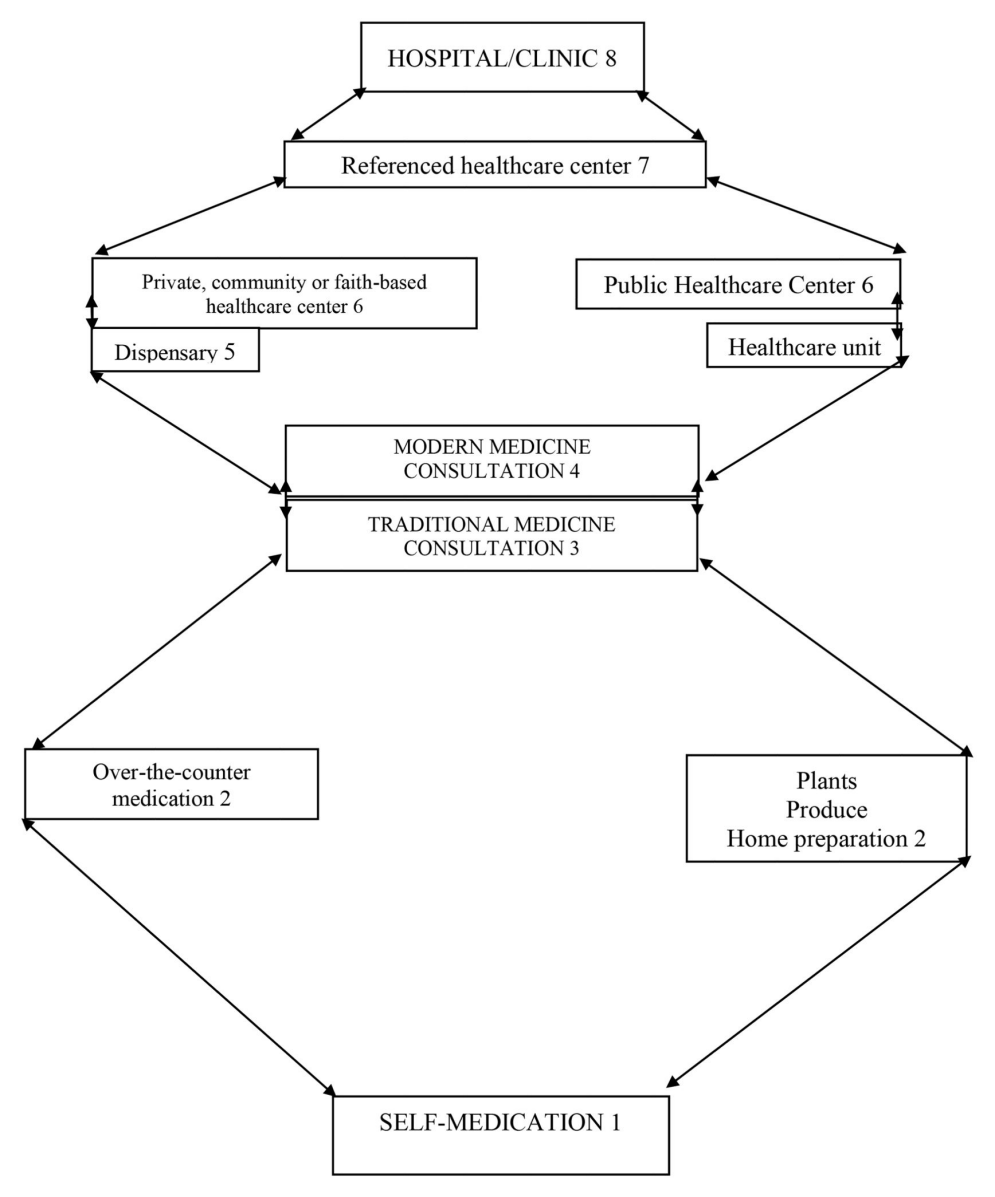
Bolen-Gourde of Care Use: Therapeutic Roaming

## BOLEN GOURDE OF CASE USE: THERAPEUTIC ITINERENCE Source: Tieman Diarra WHO Mission RDC 2019

The diagram focuses on changes from Therapeutic Itinerary to Therapeutic Itinerance. Our findings show that therapeutic roaming leads to higher transmission rates. ETCs appear to be the last resort in the therapeutic process when all other options have failed, which can be attributed to rumors about ETCs. Such misinformation jeopardizes ETCs as a viable treatment option, because people perceive them as a place for dying rather than for healing. Such perceptions concerning Western medical facilities also exist in other African communities. For example, in Sierra Leone, some people attribute EVD to a curse, a plague, or some other mystical origin^21^. Consequently, people are reluctant to seek treatment at ETCs. Rumors about EVD in the North Kivu and Ituri provinces, as well as in the Equateur province and other parts of Africa, hinder the *riposte* staff’s ability to provide support concerning the disease.

From several interviews with patients and their relatives, we found that patients used multiple remedies simultaneously, specifically, both modern and traditional medicine, and that they alternated between the two. This plurality of treatment options indicates that some went to healthcare centers, traditional healers, and religious leaders, seeking treatment^22^. The therapeutic journey/ pathway/route of those who recovered from EVD followed the same pattern of health care use. The patients’ post-recovery journeys were long, complex, and involved a rearrangement of their daily lives^22^. Often, the treatment option chosen was motivated by financial means and due to a lack of means; some community members were not able to obtain healthcare services^23^. The inferior quality of healthcare services and an inhospitable environment also cause non-attendance at healthcare facilities^23^. In some cases, it was the community’s lack of confidence in the healthcare services during the epidemic that led to the decline in their attendance^24,25^.

A nurse in Mbogu noted that in many cases, the patient does not know which treatment has been effective and led to recovery, as both traditional and modern medicines were used simultaneously. Many community members fear healthcare facilities, because they are viewed as a potential source of contracting EVD and other diseases, which then engenders a reluctance to use them^26^. This fear is created by mistrust and misinformation about healthcare facilities, and has caused people to abandon their use in the EVD context, even if they had used these facilities previously for other ailments^26^. Therefore, many community members turn to self-medication as their primary treatment option.

We examined the elements that lead to seeking treatment for a disease such as EVD. Accordingly, the precipitating event is the disease itself, but many factors affect the pattern of healthcare options used. For instance, resource availability impacts the use of healthcare services. Patients tend to seek treatment if there are services, opportunities, and providers available to them. Patients also use the information available to them from previous experiences, services, and opportunities. Therefore, we found that the decision to seek treatment depends on several elements, as opposed to just one^27^. As mentioned above, despite these elements, for some diseases, modern medicine is not considered a treatment option at all. In other words, patients do not use a healthcare center or go to any healthcare facility. In such cases, only traditional medicine and self-medication are utilized.

Self-medication is the dominant treatment option, even in the econometric analysis of the therapeutic journey/ pathway/route. After self-medication, most people resort to public services, then to modern private healthcare services and traditional medicine^28^. Therapeutic pluralism has often been described^28^, and even in the treatment of a specific disease such as malaria, people often use self-medication, healthcare facilities, and a traditional healer^29^.

Several observations arose from a cross-section of the interviews and FGDs regarding people’s first recourse to healthcare as described below.

1. Self-medication is the first treatment option chosen when people seek healthcare in their communities. When it fails, people explore other treatment options.
2. In self-medication, patients first use the resources of traditional medicine: plants, minerals, vegetables, and animal materials. Traditional healers mentioned that these elements were used in the preparation of remedies.
3. Pharmacies are part of the self-medication treatment option. People go to pharmacies without prior consultation with a medical specialist or a medical prescription.
4. Pharmacies’ response to requests for self-medication also contributes to the decision to resort to them.
5. After resorting to a certain solution, the previous recourse is not necessarily abandoned. Healthcare workers stated that some patients continued taking traditional medication while they were in healthcare centers. However, this can cause patient confusion, as they claim to not know which of the treatments was effective. Simultaneous recourse, which is part of the logic of the multiplication of chances, was also observed elsewhere.
6. The therapeutic space becomes a concentric space—an intertwined space in which the comings and goings happen between the various levels of healthcare providers.
7. Although healthcare centers (e.g., hospitals and clinics) are the last resort, they can also be gateways to the healthcare system. Some traditional healers spoke of patients who had come to them after having exhausted the possibilities of modern healthcare facilities.
8. Leaving a healthcare facility, which patients sometimes consider an escape, is not perceived negatively by patients influenced by rumors, specifically in the context of the epidemic.
9. Rumors about healthcare centers and ETCs, in the epidemic context, feed patients’ and their relatives’ fear. This contributes to the desertion of healthcare facilities or treatment abandonment in search of other treatment options.
10. Patients and their relatives perceive the search for treatment as an opportunity to try all options at once and do not always consider one possibility as the final remedy. Patients and their relatives transform their therapeutic journeys into a process of therapeutic roaming.
11. The process of therapeutic roaming is the basis of disease transmission in the context of the EVD epidemic. It overexposes healthcare providers to EVD when patients, or their relatives, resort to multiple options while confronting undiagnosed EVD.
12. Therapeutic roaming occurs among care options, but this takes place within the community. The patient and their relatives may use different means of transportation—including public transportation on land, water, and even air—therefore placing other people at risk.
13. Roaming results in the transmission of the disease to various locations. It is especially dangerous if the disease goes undetected in the new setting/area/ place.
14. When therapeutic roaming occurs after leaving an ETC, it becomes partially pathogenic. The patient may flee the ETC for other destinations based on their motivations, which are often fueled by their perceptions of EVD and its treatment, as discussed below.

Therapeutic journeys are revealed from the decisions made by the patient and their families to seek healthcare. Although some people go directly to a healthcare facility when they are ill, there is a classic itinerary that is followed by the majority of the population, as was discovered in the interviews and FGDs.

The first is self-medication at home comprising well-known traditional remedies. This is especially true when children fall ill. The term *enema* is used to describe this phenomenon. Self-medication is usually carried out with herbal medicine.

Next, the patient or their relatives visit a pharmacy to obtain a known medicine. Often, they might bring an old empty box of the medicine they wish to purchase. The patient or their family members describe the symptoms being experienced, and it is often up to the pharmacist to select what they believe to be the correct medication for the symptoms.

Finally, patients and their relatives will resort to a healthcare provider. This provider may be from the modern or traditional healthcare system. They may also choose to use both options, one after the other. It is possible that once a traditional healer has been consulted, the patient may see a modern medicine provider as well.

The use of modern medicine providers is usually done from the smallest center to the largest: healthcare unit, healthcare center, and then hospital. Usually, recourse to healthcare services occurs late in the progression of the disease. All the other possibilities are exhausted first, such that when the patient arrives at the healthcare facilities, the disease is at an advanced stage. Therefore, treatment is usually more complicated than if the healthcare facility was the first option.

## Limitations

The finding that some people immediately seek help from a healthcare center or hospital was true for about a third of the participants. We surmise that this result may have been the product of the interaction between the participants and the interviewers, who were likely to be perceived as healthcare employees. Participants’ answers may have been guided by the perceived status of their interviewers.

## Concluding remarks

Therapeutic itineraries are the result of healthcare choices. We set out to examine the pattern of healthcare treatment options used among those with EVD. We established that there are multiple choices available, and the journey involves a degree of therapeutic pluralism. We found that patients do not favor the use of healthcare facilities and prefer to begin with self-medication. Patients and their relatives first attempt to treat themselves based on what they already know about the disease and what medication they already have in their possession.

Once their resources and knowledge are exhausted, patients and their relatives usually go to a pharmacy to obtain medication. They ask for medication that they know is effective in treating the disease, asking the pharmacist for a particular medication by name or by providing an empty package of a specific medication. Sometimes, they inform the pharmacist of their symptoms for the pharmacist to recommend the appropriate medication. Vendors or pharmacists do not always require a prescription to sell medication, which also encourages people to go to pharmacies before visiting a healthcare facility. Resorting to the pharmacy as the next step in the process saves both time and money, as the patient does not need a prior consultation with a healthcare practitioner to obtain a medical prescription.

If resorting to the pharmacy does not cure the disease, then the patient or their relatives usually seek assistance from a traditional healer, and traditional and complementary medicine is as much a part of the search for a cure as modern medicine. In our study, this was confirmed by the traditional healers themselves.

When the disease cannot be treated by a traditional healer, patients or their relatives visit the nearest healthcare center. This step in patients’ therapeutic journey is affected by economy of time and money as well as a healthcare center’s proximity to the patient. Patients and their relatives visit the closest and most economical center.

The next step in the process, the referral healthcare center, is an option only if the nearest healthcare center was not able to satisfy or treat and cure the patient. This decision to take the next step can come from the patient, their relatives, or a healthcare worker. When it comes from a healthcare worker, the referral is usually made to a larger facility that will offer a greater chance of treating the disease.

Thus, a hospital is usually at the end of the healthcare alternatives chain, as it usually offers the greatest possibility of treatment and a cure. The therapeutic itinerary is also motivated by the characteristics of the disease. Some diseases are only considered treatable by traditional medicine. Many of these diseases have similar symptoms to EVD, and in such cases patients do not seek treatment from healthcare facilities. For many cured patients, traditional medicine was chosen first because they thought they had been poisoned.

The therapeutic itinerary is not linear and seeking treatment in a hospital does not exclude the possibility of consulting a traditional healer later. This recourse is often made simultaneously—from modern medicine to traditional medicine to self-medication. As such, the therapeutic itinerary becomes therapeutic roaming. Patients leave the treatment center for other treatment possibilities because they want to try everything. In other words, therapeutic roaming is believed to increase the chances of recovery. However, in the case of EVD, therapeutic roaming also increases the possibilities of transmitting the disease. The same applies to a therapeutic itinerary, which exposes the patient, their relatives, and healthcare providers (whether modern or traditional medicine practitioners).

## Figures and Tables

**Figure 3:**
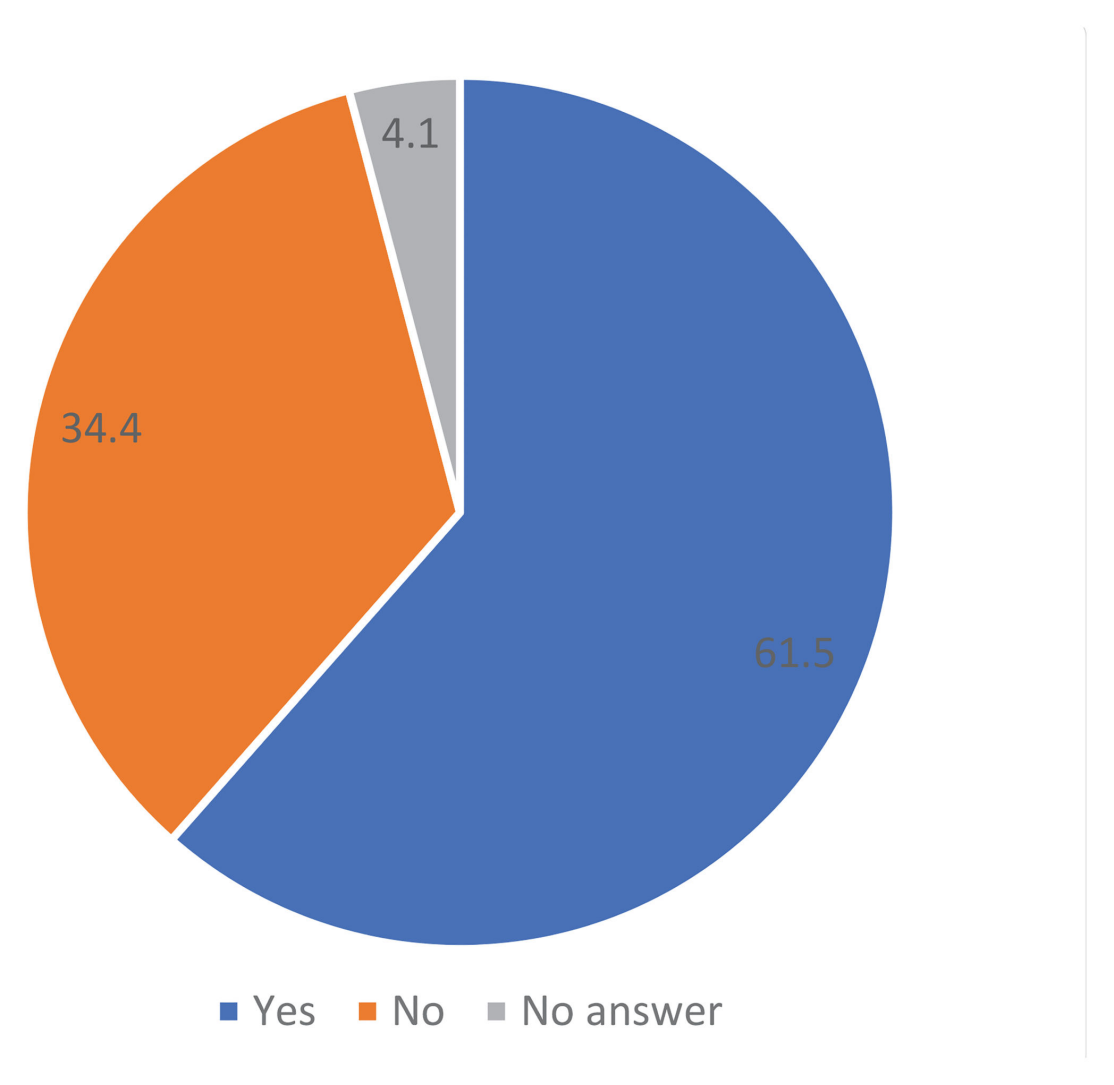
Existence of diseases for which modern medicine is not considered a treatment option

**Table 1:** Distribution of participants in the in-depth interviews and focus group discussions by province

Target	North Kivu Province				Ituri Province			
	Butembo		Beni		Mbuti		Bunia	
	IDI	FGD	IDI	FGD	IDI	FGD	IDI	FGD
Pillar leads	All		All		All		All	
Pillar members	2/pillar		2/pillar		2/pillar		2/pillar	
Community leaders^1^	≥2/ community		≥2/ community		≥2/ community		≥2/ community	
Leader of survivor group	≥2/ community		≥2/ community		≥2/ community		≥2/ community	
Community adult males		≥2 groups		≥2 groups		≥2 groups		≥2 groups
Community adult females		≥2 groups		≥2 groups		≥2 groups		≥2 groups
Community male youth		≥2 groups		≥2 groups		≥2 groups		≥2 groups
Community female youth		≥2 groups		≥2 groups		≥2 groups		≥2 groups
Survivors		≥2 groups		≥2 groups		≥2 groups		≥2 groups

1 The community leaders, here, include traditional, political, religious, and opinion leaders in the community

## Data Availability

The data supporting the findings of this study are not publicly available because they contain information that could compromise the privacy of the research participants. However, the data are available from the corresponding author (Joseph Okeibunor) upon reasonable request.
